# G-RANK: an equivariant graph neural network for the scoring of protein–protein docking models

**DOI:** 10.1093/bioadv/vbad011

**Published:** 2023-02-03

**Authors:** Ha Young Kim, Sungsik Kim, Woong-Yang Park, Dongsup Kim

**Affiliations:** Department of Bio and Brain Engineering, Korea Advanced Institute of Science and Technology, Daejeon 34141, South Korea; GENINUS Inc., Seoul 05836, South Korea; GENINUS Inc., Seoul 05836, South Korea; Samsung Genome Institute, Samsung Medical Center, Seoul 06351, South Korea; Department of Molecular Cell Biology, Sungkyunkwan University School of Medicine, Suwon 16419, South Korea; Department of Bio and Brain Engineering, Korea Advanced Institute of Science and Technology, Daejeon 34141, South Korea

## Abstract

**Motivation:**

Protein complex structure prediction is important for many applications in bioengineering. A widely used method for predicting the structure of protein complexes is computational docking. Although many tools for scoring protein–protein docking models have been developed, it is still a challenge to accurately identify near-native models for unknown protein complexes. A recently proposed model called the geometric vector perceptron–graph neural network (GVP-GNN), a subtype of equivariant graph neural networks, has demonstrated success in various 3D molecular structure modeling tasks.

**Results:**

Herein, we present G-RANK, a GVP-GNN-based method for the scoring of protein-protein docking models. When evaluated on two different test datasets, G-RANK achieved a performance competitive with or better than the state-of-the-art scoring functions. We expect G-RANK to be a useful tool for various applications in biological engineering.

**Availability and implementation:**

Source code is available at https://github.com/ha01994/grank.

**Contact:**

kds@kaist.ac.kr

**Supplementary information:**

Supplementary data are available at *Bioinformatics Advances* online.

## 1 Introduction

Protein–protein interactions play key roles in many biological processes. Knowledge on the 3D structures of protein complexes is key for understanding protein–protein interactions, which can be useful in various applications such as protein design or drug discovery. As experimental approaches pose limitations in terms of time and cost, computational docking is commonly used to predict the structures of protein complexes. Computational docking involves the generation of a large number of candidate structural models, from which final models are selected according to a certain scoring function. The scoring function is highly important for the successful identification of near-native docking models among all generated candidates.

Various scoring functions have been developed based on physics-based, statistical potential-based, or machine learning-based methods (Geng *et al.*, 2020). Examples of physics-based methods include HADDOCK (Dominguez *et al.*, 2003), ZDock (Pierce *et al.*, 2014) and pyDock (Cheng *et al.*, 2007). Examples of methods based on knowledge-based statistical potentials include GOAP (Zhou and Skolnick, 2011) and ITScore (Huang and Zou, 2008). Finally, machine learning-based methods include iScore (Geng *et al.*, 2020), DeepRank (Renaud *et al.*, 2021), DOVE (Wang *et al.*, 2020), GNN-DOVE (Wang *et al.*, 2021), PAUL (Eismann *et al.*, 2021) and DeepRank-GNN (Réau *et al.*, 2023). Currently, there is no comprehensive benchmarking study on the performances of the more recently developed machine learning methods. However, these machine learning-based methods show high predictive performances in their own assessment studies, often outperforming the traditional scoring functions.

The models introduced above have achieved considerable success in scoring protein-protein docking models, but there is still room for improvement (Réau *et al.*, 2023). Recently, a new type of neural network called the equivariant graph neural network (EGNN) has been proposed (Satorras *et al.*, 2021). If a function is equivariant to some transformation, then the transformation of the input results in an equivalent transformation of the function’s output (Townshend *et al.*, 2021). EGNNs are E(3)-equivariant; that is, they are equivariant to translation, rotation and reflection in 3D Euclidean space (Satorras *et al.*, 2021). Equivariance is a desired property because neural networks are needed to recognize proteins in different positions and orientations. A subtype of EGNN, geometric vector perceptron–graph neural network (GVP-GNN), has recently shown success in various 3D molecular structure modeling tasks (Jing *et al.*, 2020, 2021).

Herein, we propose G-RANK, a new GVP-GNN-based method, for ranking protein–protein docking models. We compare our model with DeepRank-GNN (Réau *et al.*, 2023), GNN-DOVE (Wang *et al.*, 2021), iScore (Geng *et al.*, 2020), HADDOCK (Van Zundert *et al.*, 2016) and GOAP (Zhou and Skolnick, 2011). When tested on two different test datasets, our model’s performance is competitive with or better than the compared scoring functions. We expect this model to be a useful tool for predicting the structures of protein–protein complexes.

## 2 Materials and methods

### 2.1 Model architecture

We used the GVP-GNN model architecture from Jing *et al.* (2021). GVP-GNNs differ from typical EGNNs in that the node and edge embeddings are tuples of scalar and vector features. Multi-layer perceptrons in standard EGNNs are replaced with GVPs in GVP-GNNs. A GVP is a module with a scalar channel and a vector channel that can process scalar and vector features, respectively. This module involves information propagation from a scalar channel to a vector channel and vice versa. Details on the formulation of GVP can be found in the original article (Jing *et al.*, 2021).

**Fig. 1.**
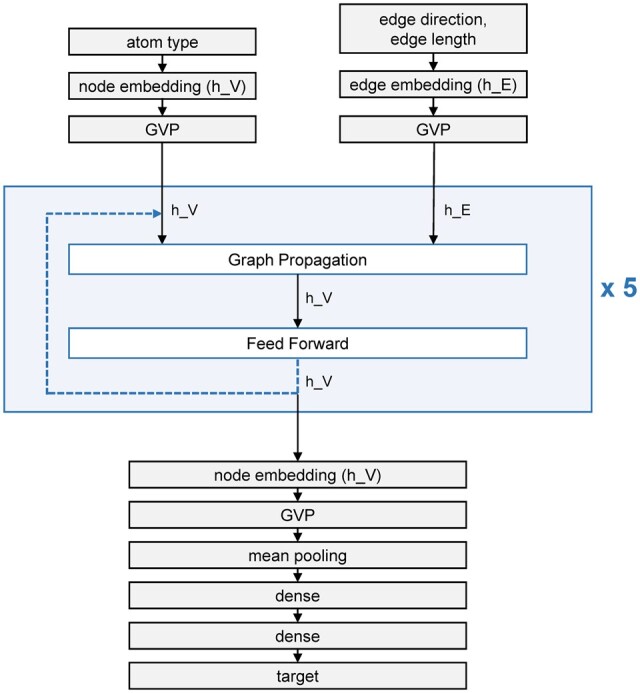
Architecture of GVP-GNN developed by Jing *et al.* (2021). GVPConvLayer block is shown in blue. Node embedding is indicated by h_V, and edge embedding is indicated by h_E

The architecture of the model is shown in Figure 1. Nodes are defined as atoms, and edges are defined for all pairs of atoms within 4.5 Å. The initial node embedding is the atom type in one-hot encoding. The initial edge embedding consists of the Gaussian radial basis function encoding of the edge length and the unit vector in the edge direction. These edge embeddings are computed from the atom coordinates. After the node and edge embeddings are each passed through a GVP layer, they are provided as input to a GVPConvLayer block which is repeated five times. This block consists of two layers: a graph propagation layer and a feed-forward layer.

The graph propagation layer first computes messages from node and edge embeddings and then uses the message to update node embeddings. This layer can be formulated as:

(1) $$\begin{array}{c} m^{\left(j\to i\right)}=\;\phi \left(\mathrm{concat}\left(h_{V}^{\left(j\right)},h_{E}^{\left(j\to i\right)}\right)\right)\;\mathrm{for}\;j^{'}s\;\mathrm{in}\;N\left(i\right)\\ h_{V}^{\left(i\right)}\leftarrow \mathrm{LayerNorm}\left(h_{V}^{\left(i\right)}+\;\frac{1}{k^{'}}\mathrm{Dropout}\left(\sum_{j\in N(i)}m^{\left(j\to i\right)}\right)\right), \end{array}$$

where $m^{\left(j\to i\right)}$ is the message from node j to node i, ϕ is a sequence of 3 GVP layers, $h_{V}^{\left(j\right)}$ is the embedding of node j, $h_{E}^{\left(j\to i\right)}$ is the embedding of edge $\left(j\to i\right)$, N(i) is the set of neighboring nodes of node i, and $k^{\prime }$ is the size of N(i).

The feed-forward layer then updates node embeddings in a point-wise manner. This layer can be formulated as:

(2) $$h_{V}^{\left(i\right)}\leftarrow \mathrm{LayerNorm}\left(h_{V}^{\left(i\right)}+\mathrm{Dropout}\left(\phi \left(h_{V}^{\left(i\right)}\right)\right)\right),$$

where ϕ is a sequence of 2 GVP layers. The updated node embeddings are repeatedly used as the input node embeddings for the next GVPConvLayer block.

After five repetitions of the GVPConvLayer block, the final node embeddings are passed through a GVP layer, after which all node embeddings are reduced to scalars. This is followed by a mean pooling layer that averages the embeddings across all nodes. The embeddings finally pass through two dense layers, thus resulting in a single scalar value.

### 2.2 Datasets

#### 2.2.1 BM5 cross-validation dataset

The authors of DeepRank (Renaud *et al.*, 2021) conducted docking by using HADDOCK version 2.2 (Dominguez *et al.*, 2003; Van Zundert *et al.*, 2016) on the docking benchmark dataset version 5 (BM5) (Vreven *et al.*, 2015). The authors of DeepRank obtained 232 non-redundant complexes from the BM5 dataset, from which they excluded antibody–antigen complexes and complexes involving more than two chains, which resulted in 142 complexes. The dataset was made available by the authors of DeepRank (available at https://data.sbgrid.org/dataset/843/), and the details on the dataset generation can be found in Supplementary Note S1. The dataset was used by the authors of DeepRank-GNN (Réau *et al.*, 2023) for 10-fold cross-validation. The cross-validation is performed in the following manner: 15 complexes are left out as the test set. For each fold, 102 and 25 complexes are assigned to the training and validation sets, respectively. We use the same cross-validation dataset as DeepRank-GNN to ensure a fair comparison. Details on the size and composition of the 10-fold cross-validation dataset can be found in Supplementary Table S2.

#### 2.2.2 Independent test dataset

We assessed our model on an independent test dataset called the CAPRI score set (Lensink and Wodak, 2014), which was also made available by the authors of DeepRank (available at https://data.sbgrid.org/dataset/843/). This dataset consists of more than 16 000 docking models for 13 protein–protein complexes, which were generated using a variety of docking methods by more than 40 research groups. We left out one complex containing no positive samples, resulting in 12 complexes. Details on the size and composition of this dataset can be found in Supplementary Table S3.

### 2.3 Model inputs and training

As in DeepRank-GNN (Réau *et al.*, 2023), we constructed the graph only on the interface of the protein–protein complex, rather than the full complex. The interface was determined with pdb2sql (Renaud and Geng, 2020) at a cutoff of 8.5 Å. Some models for which no contact atoms were found were excluded. Five atoms, C, N, O, S and H, were included in the model inputs. The ATOM3D package (Townshend *et al.*, 2020) was used to convert the PDB files into the LMDB data format required for model inputs.

We trained G-RANK to predict the *f-nat* (fraction of native contacts) (Méndez *et al.*, 2003) values of docking models. *f-nat* ranges from 0 to 1, with higher *f-nat* values indicating higher model quality. The cutoff for discriminating between near-native and wrong models was defined as 0.3, as done in DeepRank-GNN (Réau *et al.*, 2023). The mean squared error was used as the loss function, and Adam optimizer was used for optimization. The models were trained by using a batch size of 64 and a learning rate of 0.0001. We trained the models for 50 epochs, saved the model in each epoch and retained the model with the lowest validation loss for final evaluation on the test set.

### 2.4 Assessment metrics

We used the area under the receiver operating characteristic curve (ROC-AUC) and the precision-recall area under the curve (PR-AUC) to assess model performance. We also used a per-complex assessment metric called the hit rate (Renaud *et al.*, 2021), which is defined as follows:

(3) $$\mathrm{hit}\;\mathrm{rate}\left(k\right)=\;\frac{N_{\mathrm{hits}}(k)\;}{N_{\mathrm{pos}}},$$

where $N_{\mathrm{hits}}(k)$ is the number of hits (positive models) among the top k ranked models predicted by the predictor, and $N_{\mathrm{pos}}$ is the total number of positive models for this complex. We also assess using the success rate, which is the number of docking cases with at least one near-native model among the top-*k* predicted models, divided by the total number of cases.

## 3 Results

### 3.1 Results on the BM5 test set

**Fig. 2.**
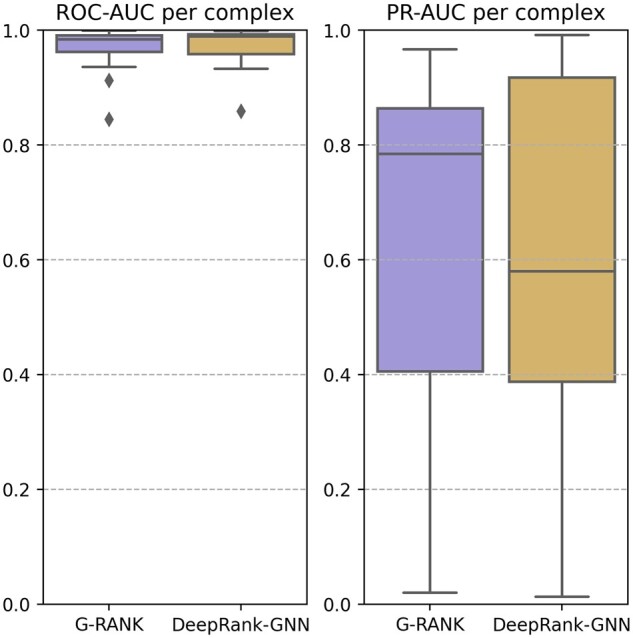
Distributions of per-complex ROC-AUC and PR-AUC on the BM5 test set

**Fig. 3.**
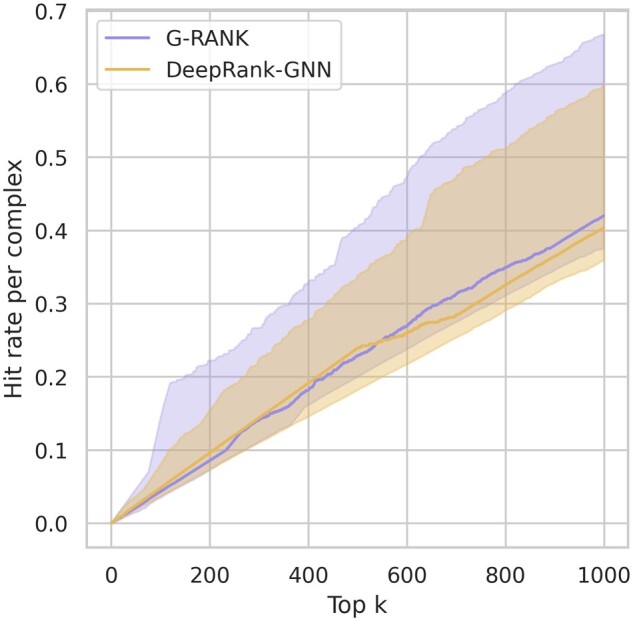
Distributions of per-complex hit rates on the BM5 test set, where *k* ranges from 0 to 1000. The thick lines denote the median hit rates, and the shaded areas denote the 25–75% quantile interval of the hit rates

We trained G-RANK on the BM5 10-fold cross-validation dataset (training and validation loss curves shown in Supplementary Fig. S4). Afterwards, we used an ensemble method to compare the performance of the model with that of DeepRank-GNN by averaging the predictions of all models from the 10 folds for final evaluation on the left-out test set. DeepRank-GNN predictions were also obtained using the same ensemble method. The left-out test set consisted of 15 complexes with 22 099 positive models and 342 096 negative models. The boxplots in Figure 2 show the distributions of per-complex ROC-AUC values and per-complex PR-AUC values. Both G-RANK and DeepRank-GNN tended to achieve high ROC-AUC values. In terms of PR-AUC, G-RANK achieved a lower 75% quantile than DeepRank-GNN (0.864 < 0.918) but a significantly higher median value (0.785 > 0.581). Figure 3 shows the per-complex hit rates for the top-*k* ranked models, for values of *k* ranging from 0 to 1000. The performance of G-RANK is similar to that of DeepRank-GNN in terms of the median but outperforms DeepRank-GNN in terms of the 75% quantile.

### 3.2 Results on the independent test dataset

**Fig. 4.**
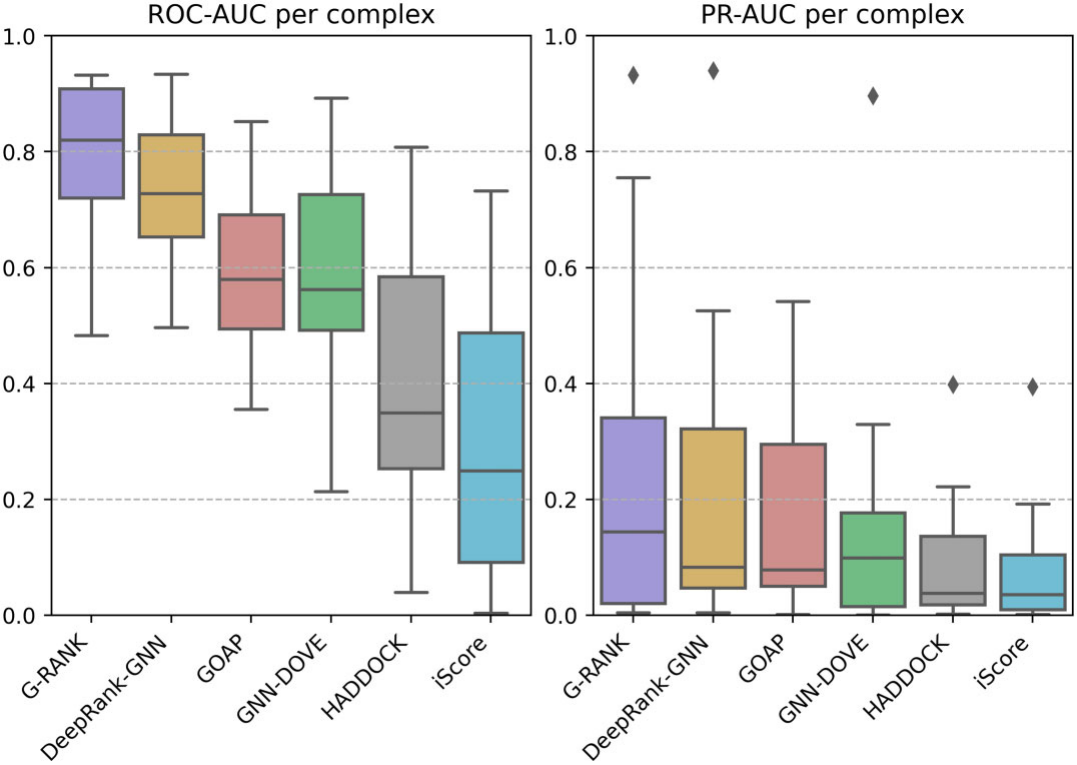
Distributions of per-complex ROC-AUC and PR-AUC on the CAPRI score set

**Fig. 5.**
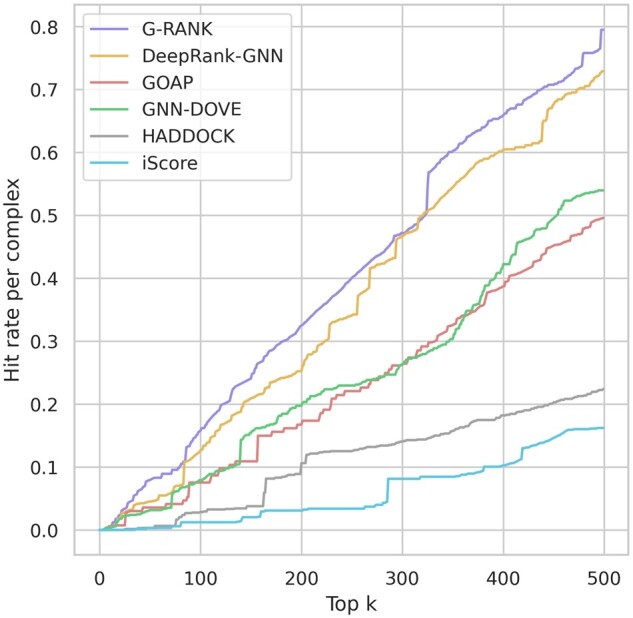
Distributions of the median of the per-complex hit rates on the CAPRI score set, where *k* ranges from 0 to 500

**Fig. 6.**
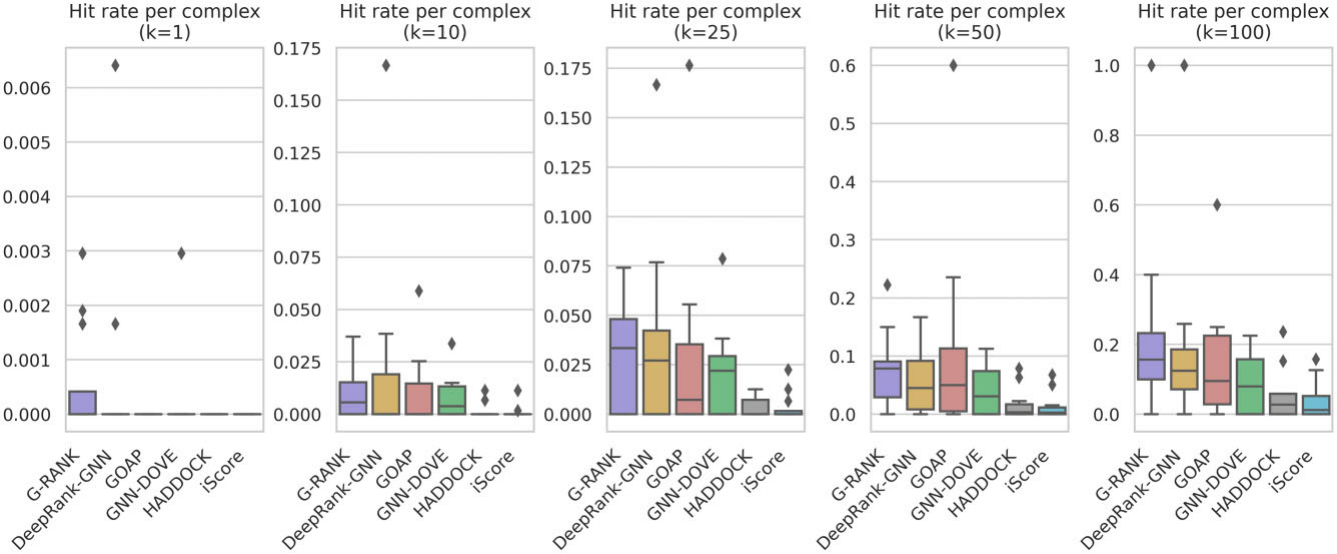
Distributions of per-complex hit rates on the CAPRI score set (*k *=* *1, 10, 25, 50, 100)

We used the ensemble method described above to evaluate our model on the CAPRI score set consisting of 1970 positive models and 14 113 negative models. We compared our model with DeepRank-GNN, GOAP, GNN-DOVE, HADDOCK and iScore. Figure 4 shows the distributions of per-complex ROC-AUC and PR-AUC values. In terms of both metrics, G-RANK achieved a higher distribution of scores than other methods. Figure 5 shows the median of per-complex hit rates for the top-*k* ranked models, for values of *k* ranging from 0 to 500, and Figure 6 shows the distributions of per-complex hit rates for *k = *1, 10, 25, 50, 100. In both figures, it can be seen that the median hit rates of G-RANK are overall higher compared to other methods. Also, Table 1 shows the success rates when *k = *1, 10, 25, 50, 100. G-RANK demonstrates the highest success rate among all predictors for values of *k = *1, 10, 25, 50.

**Table 1.** Assessment of success rates on the CAPRI score set (*k *=* *1, 10, 25, 50, 100)

	G-RANK	DeepRank-GNN	GOAP	GNN-DOVE	HADDOCK	iScore
Top 1	**0.25**	0.17	0.00	0.08	0.00	0.00
Top 10	**0.5**	0.42	0.42	**0.5**	0.17	0.17
Top 25	**0.58**	**0.58**	**0.58**	**0.58**	0.42	0.25
Top 50	**0.75**	**0.75**	**0.75**	0.58	0.5	0.5
Top 100	0.83	**0.92**	0.83	0.67	0.58	0.58

*Note*: Success rate refers to the percentage of cases in which a correct model is found within the top *k* ranked models.

Additionally, we performed a model assessment by labeling the correct and incorrect models using the CAPRI scoring criteria (Lensink *et al.*, 2018), instead of *f-nat* values. CAPRI classes are computed based on iRMSD (interface RMSD), lRMSD (ligand RMSD) and *f-nat* values, and each docking model is classified as ‘high’, ‘medium’, ‘acceptable’ or ‘incorrect’ (Supplementary Note S5). In this assessment, we consider a model correct if it is at least of ‘acceptable’ quality. The per-complex ROC-AUC, PR-AUC, hit rates and the success rates are shown in Supplementary Figures S6 and S7 and Supplementary Table S8. In terms of ROC-AUC and PR-AUC, G-RANK still achieved the highest performance. In terms of the hit rates, when the value of *k* is small, it shows a slightly lower performance compared to other models, but it generally shows a good predictive performance. In terms of the success rates, its performance is similar to other methods in general.

## 4 Discussion

In this study, we developed G-RANK, a new method based on GVP-GNN for assessing the quality of protein–protein docking models. When tested on two different test sets, G-RANK’s performance was competitive with or outperformed the existing state-of-the-art methods. Analysis on hit rates and success rates demonstrated that G-RANK performs well in identifying near-native models among a large pool of candidate models. Our results confirm the previous finding (Jing *et al.*, 2021) that GVP-GNNs perform well across prediction tasks involving 3D molecular structures. The equivariance property of the neural network, as well as the representation of 3D structures as both scalar and vectors, is important factors that led to G-RANK’s outstanding performance.

As shown by the generally low PR-AUC of the predictors on the CAPRI score set, the identification of several near-native models among a large pool of candidate models remains a difficult problem. Development of more advanced equivariant neural network architectures in the future may help overcome this problem. In addition, the continual accumulation of experimentally determined protein complex structures may enable better prediction in the future. Also, the recent development of AlphaFold2-Multimer (Evans *et al.*, 2022), a fold-and-dock approach, has shown significant success in the prediction of protein complex structures. We expect that the scoring function developed in this work can collaborate with such model to help advance our knowledge on protein complex structures.

The GVP-GNN model used in this study could be further modified and applied to other research objectives related to protein structure and function, such as ranking of protein–ligand docking models. We expect that G-RANK may contribute to various applications in biological engineering, such as protein design and drug discovery.

## Supplementary Material

vbad011_Supplementary_Data

## Data Availability

Data is made available by the authors of DeepRank and can be accessed at https://data.sbgrid.org/dataset/843/.
