# Supplementary material for: G-RANK: an equivariant graph neural network for the scoring of protein–protein docking models
Source: Bioinform Adv. 2023 Feb 3;3(1):vbad011. doi: 10.1093/bioadv/vbad011 (PMC9927558; doi:10.1093/bioadv/vbad011)
Supplement: vbad011_Supplementary_Data [file vbad011_supplementary_data.docx]

**Supplementary Information for:**

**An Equivariant Graph Neural Network for assessing the quality of structural models of protein complexes**

**Ha Young Kim^1^, Sungsik Kim^2^, Woong-Yang Park^2,3,4^, Dongsup Kim^1,*^**

^1^Department of Bio and Brain Engineering, Korea Advanced Institute of Science and Technology, Daejeon 34141, South Korea

^2^GENINUS Inc., Seoul, South Korea

^3^Samsung Genome Institute, Samsung Medical Center, Seoul, South Korea

^4^Deparment of Molecular Cell Biology, Sungkyunkwan University School of Medicine, Suwon, South Korea

*Corresponding author ([kds@kaist.ac.kr](mailto:kds@kaist.ac.kr))

Table of Contents

[**Supplementary Note S1.** Details on the generation of the HADDOCK docking models by the authors of DeepRank 1](#_Toc125209424)

[**Supplementary Table S2.** Size and composition of the BM5 cross validation dataset used in this study 2](#_Toc125209425)

[**Supplementary Table S3.** Size and composition of the CAPRI score set 3](#_Toc125209426)

[**Supplementary Figure S4.** Training and validation loss curves and validation ROC-AUC curves from the 10 fold cross validation. 4](#_Toc125209427)

[**Supplementary Note S5.** Details on the CAPRI scoring criteria 7](#_Toc125209428)

[**Supplementary Figure S6.** Assessment of per-complex ROC-AUC and PR-AUC on the CAPRI score set, where the discrimination of correct and incorrect models is based on the CAPRI scoring criteria. 8](#_Toc125209429)

[**Supplementary Figure S7.** Assessment of per-complex hit rates on the CAPRI score set, where the discrimination of correct and incorrect models is based on the CAPRI scoring criteria. 8](#_Toc125209430)

[**Supplementary Table S8.** Assessment of success rates on the CAPRI score set, where the discrimination of correct and incorrect models is based on the CAPRI scoring criteria. 9](#_Toc125209431)

### **Supplementary Note S1.** Details on the generation of the HADDOCK docking models by the authors of DeepRank

The HADDOCK docking models of the BM5 dataset were generated by the authors of DeepRank (Renaud, et al., 2021) and also used by the authors of DeepRank-GNN (Réau, et al., 2022). The authors of DeepRank obtained 232 non-redundant complexes from the BM5 dataset, from which they excluded antibody-antigen complexes and complexes involving more than two chains, which resulted in 142 complexes.

According to the authors of DeepRank-GNN, the generation of docking models with HADDOCK was conducted under the following five different scenarios:

1) docking with random surface patch restraints (10000/400/400 models for it0/it1/water stages)

2) docking with center of mass restrains (10000/400/400 models for it0/it1/water stages)

3) docking with true interface residues defined within a 5 A distance to the partner protein (1000/400/400 for it0/it1/water stages)

4) docking with true interface residues defined within a 3.9 A distance to the partner protein (1000/400/400 for it0/it1/water stages)

5) refinement of the bound complex (50/50 it1/water stages)

All scenarios, except for scenario 5, are docking with unbound conformations. Docking with bound conformations constitute a small proportion (100 models out of 25300 models per complex).

### **Supplementary Table S2.** Size and composition of the BM5 cross validation dataset used in this study

|  | Train (total) | Train (positive) | Train (negative) | Valid (total) | Valid (positive) | Valid (negative) |
| --- | --- | --- | --- | --- | --- | --- |
| fold1 | 258060 | 13297 | 244763 | 63250 | 3771 | 59479 |
| fold2 | 258060 | 15032 | 243028 | 63250 | 3700 | 59550 |
| fold3 | 258060 | 18001 | 240059 | 63250 | 3052 | 60198 |
| fold4 | 258060 | 17347 | 240713 | 63250 | 5235 | 58015 |
| fold5 | 258060 | 17232 | 240828 | 63250 | 4658 | 58592 |
| fold6 | 258060 | 16730 | 241330 | 63250 | 3949 | 59301 |
| fold7 | 258060 | 17255 | 240805 | 63250 | 3917 | 59333 |
| fold8 | 258060 | 18168 | 239892 | 63250 | 4602 | 58648 |
| fold9 | 258060 | 18983 | 239077 | 63250 | 5367 | 57883 |
| fold10 | 258059 | 25580 | 232479 | 63250 | 4938 | 58312 |

| Test  (total) | Test (positive) | Test (negative) |
| --- | --- | --- |
| 364195 | 22099 | 342096 |

### **Supplementary Table S3.** Size and composition of the CAPRI score set

| CAPRI target | Total | Number of positives | Number of negatives |
| --- | --- | --- | --- |
| Target29 | 1979 | 156 | 1823 |
| Target30 | 1095 | 1 | 1094 |
| Target32 | 599 | 12 | 587 |
| Target37 | 1360 | 79 | 1281 |
| Target39 | 1287 | 5 | 1282 |
| Target40 | 1971 | 534 | 1437 |
| Target41 | 1096 | 341 | 755 |
| Target46 | 1570 | 80 | 1490 |
| Target47 | 1015 | 602 | 413 |
| Target50 | 1447 | 89 | 1358 |
| Target53 | 1360 | 54 | 1306 |
| Target54 | 1304 | 17 | 1287 |
| **TOTAL** | **16083** | **1970** | **14113** |

### **Supplementary Figure S4.** Training and validation loss curves and validation ROC-AUC curves from the 10 fold cross validation.


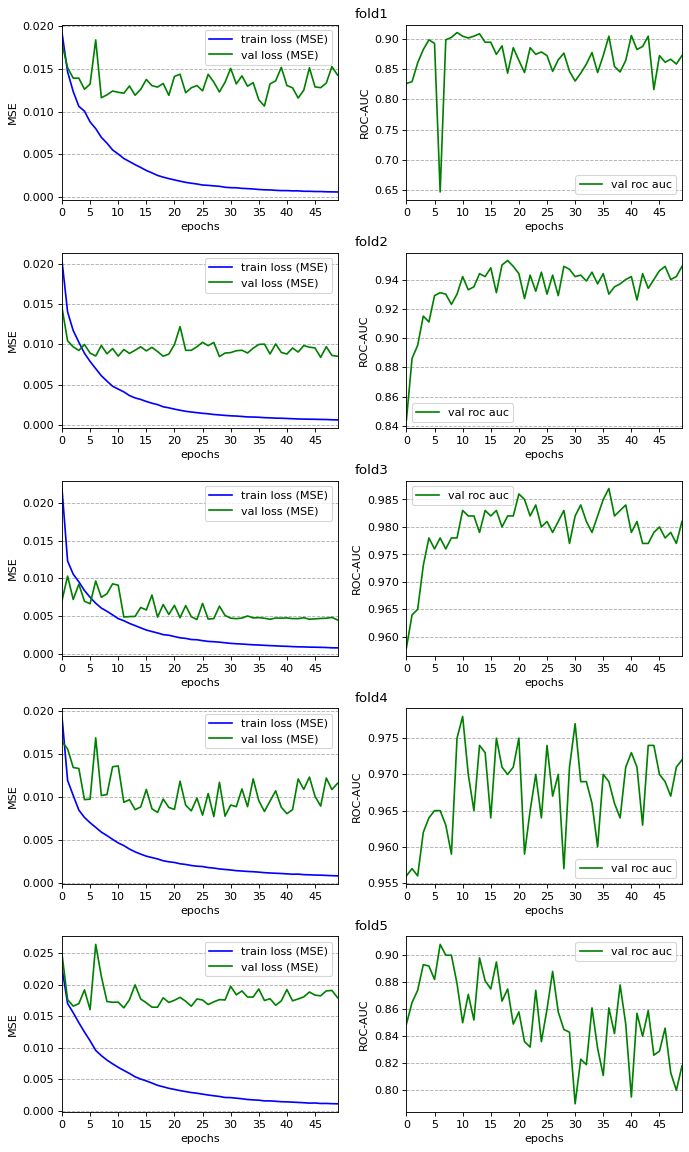


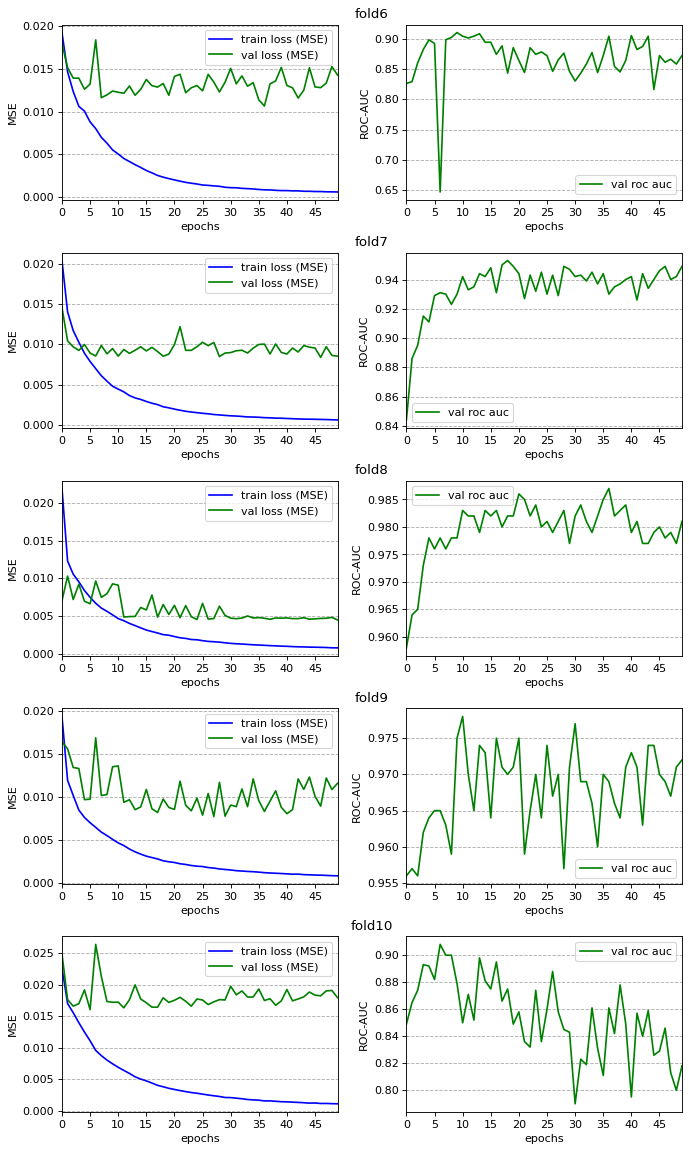


### **Supplementary Note S5.** Details on the CAPRI scoring criteria

The CAPRI scoring criteria is determined based on iRMSD (interface RMSD), lRMSD (ligand RMSD), and f-nat (fraction of native contacts) values (Lensink, et al., 2018). The quality of a decoy is assessed using the following criteria:

**Incorrect**:

fnat < 0.1 or (lrmsd > 10.0 and irmsd > 4.0)

**Acceptable**:

0.1 <= fnat < 0.3 and (lrmsd <= 10.0 or irmsd <= 4.0) or

(fnat >= 0.3 and lrmsd > 5.0 and irmsd > 2.0)

**Medium**:

0.3 <= fnat < 0.5 and (lrmsd <= 5.0 or irmsd <= 2.0) or

(fnat >= 0.5 and lrmsd > 1.0 and irmsd > 1.0)

**High**:

fnat >= 0.5 and (lrmsd <= 1.0 or irmsd <= 1.0)

### **Supplementary Figure S6.** Assessment of per-complex ROC-AUC and PR-AUC on the CAPRI score set, where the discrimination of correct and incorrect models is based on the CAPRI scoring criteria.


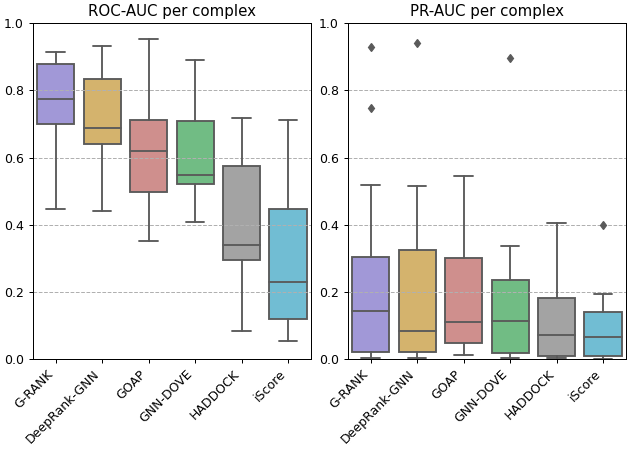


### **Supplementary Figure S7.** Assessment of per-complex hit rates on the CAPRI score set, where the discrimination of correct and incorrect models is based on the CAPRI scoring criteria.


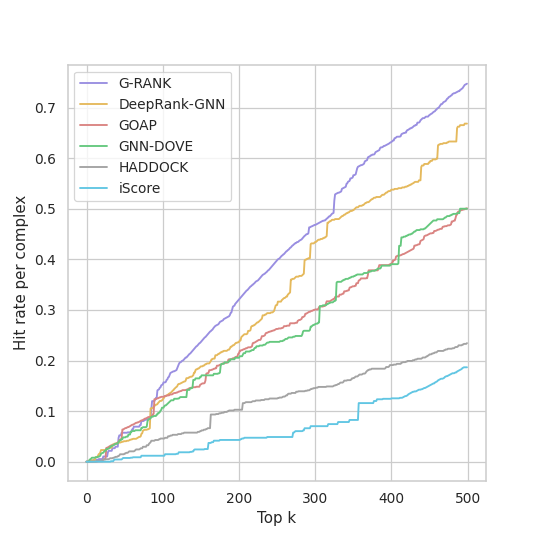


### **Supplementary Table S8.** Assessment of success rates on the CAPRI score set, where the discrimination of correct and incorrect models is based on the CAPRI scoring criteria.

|  | G-RANK | DeepRank-GNN | GOAP | GNN-DOVE | HADDOCK | iScore |
| --- | --- | --- | --- | --- | --- | --- |
| Top 1 | 0.25 | 0.25 | 0 | 0.17 | 0.08 | 0.08 |
| Top 10 | 0.5 | 0.5 | 0.33 | 0.58 | 0.25 | 0.17 |
| Top 25 | 0.5 | 0.58 | 0.58 | 0.67 | 0.58 | 0.33 |
| Top 50 | 0.67 | 0.67 | 0.75 | 0.67 | 0.67 | 0.58 |
| Top 100 | 0.83 | 0.75 | 0.83 | 0.75 | 0.67 | 0.58 |

**References**

Lensink, M.F.*, et al.* The challenge of modeling protein assemblies: the CASP12‐CAPRI experiment. *Proteins: Structure, Function, and Bioinformatics* 2018;86:257-273.

Réau, M.*, et al.* DeepRank-GNN: A Graph Neural Network Framework to Learn Patterns in Protein-Protein Interfaces. *Bioinformatics* 2022.

Renaud, N.*, et al.* DeepRank: a deep learning framework for data mining 3D protein-protein interfaces. *Nature communications* 2021;12(1):1-8.
